# Supplementary material for: Airway gene-expression classifiers for respiratory syncytial virus (RSV) disease severity in infants
Source: BMC Med Genomics. 2021 Feb 25;14:57. doi: 10.1186/s12920-021-00913-2 (PMC7908785; doi:10.1186/s12920-021-00913-2)
Supplement: Supplementary file 1 — Additional file 1: Additional technical details of RNA-Seq pre-processing, predictive model developing and cross-validation, full lists of genes selected in NGSS1 and NGSS2, and some supplemental results related to CrossNorm. [file 12920_2021_913_MOESM1_ESM.pdf]

Supplementary Methods

Removing Batch-effects in Nasal Samples

Because the study spanned three winter seasons (October 2012 through April 2015) samples were processed in six library batches. This resulted in significant batch effects in total number of mapped reads (Supplementary Figure E1). In addition, analysis of variance (ANOVA) *F*-test with false discovery rate (FDR) controlled at 0.05 level, found 3,984 genes (28.8% of the reported transcriptome) had significantly different mean expressions across batches. Based on these observations, we applied ComBat to remove batch effects. After applying ComBat, none of the genes had significant batch effects based on ANOVA *F*-test, and pairwise correlation analysis showed that the average Pearson correlation between the original and ComBat processed data was 0.987. This suggests that ComBat only removed batch effects with minimum impact on the remaining information.

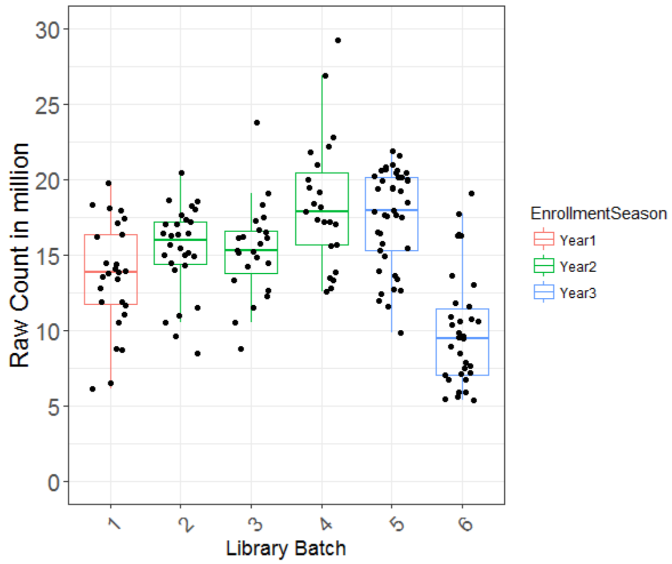

Supplementary Figure E1. Relationship between the total number of mapped reads, enrollment years, and batches in library preparation.

Model Developing and Cross-validation

***Identification of supplementary genes*.**

Both NGSS1 and NGSS2 were developed from top correlates (genes with significant marginal correlation with the GRSS) and some supplementary genes that contain information complementary to those top correlates. Specifically, we first performed principal component analyses (PCA) based on the decomposition of the correlation matrix of the top correlates (66 genes for NGSS1, and 44 genes for NGSS2), and used the leading principal component (PC1) to represent the collective information of those top correlates. Next, we conducted regression analyses with gene expressions as the response variables and the PC1 from the PCA as the covariate ($X_{ij} = \beta_{i0} + \mathrm{PC1} \cdot \beta_{i1} + \epsilon_{ij}$) for all genes except the 66 top correlates and recorded the residuals as $R_i = X_{ij} - \hat{\beta}_{i0} - PC1 \cdot \hat{\beta}_{i1}$. Here X_{ij} was the expression of the ith gene sampled from the jth subject, and R_i represented the information in X_{ij} that was uncorrelated with PC1. Finally, we computed ρ_i, the Pearson correlation between R_i and the GRSS, and select those genes with the largest $|\rho_i|$ as the supplementary genes (10 for NGSS1 and 5 for NGSS2).

Model selection strategies.

Two model selection strategies were used for developing predictive models based on multivariate regression analyses in which the GRSS was the response variable and genes were predictors. Visit age and days since disease onset were also included as candidate features. The first and primary strategy was a combination of stepwise model selection and an exhaustive search of a subset of all combinations of covariates. The second strategy is based on the elastic-net regularized regression (1, 2) and parameter refinement (3-5) based on the ordinary least-squares (OLS) criterion.

Strategy 1. We applied bi-directional stepwise model selection based on Akaike Information Criterion (AIC) to select an initial model. To further reduce model complexity, we selected a subset of *least informative* genes, defined as those with the smallest absolute values of the regression *t*-statistics in the initial model, and exhaustively searched the best sub-model of the initial model that do not include some of these 10 least informative genes (a total of 1023 combinations). Here the "best" sub-model was defined as the one with the smallest cross-validated residual sums of squares (CVRSS).

Strategy 2. As an alternative, we tried another model selection procedure based on regularized regression. Specifically, we first applied the elastic-net regularized regression, which uses both L^1 (LASSO) and L^2 (ridge) regression to produce a sparse regression model. The R package `glmnet` (2, 6) was used for this purpose. Regularization parameters were selected by an initial ten-fold cross-validation. After we obtain a sparse regression model, we re-estimate linear coefficients by OLS-based procedures to improve the accuracy of modeling fitting. The parameter refinement strategy can improve the prediction accuracy and was widely used in high-throughput data analysis (3-5).

Based on the empirical evidences in our study, we found that that Strategy 1 worked better than Strategy 2 for both NGSS1 and NGSS2, so we decided to use Strategy 1 for model selection and reported results based on Strategy 1 in the main text. A detailed comparison between these two strategies is provided in the following summary table.

Supplementary Table E1. Comparing the performance of two model selection strategies. For both NGSS1 and NGSS2, Strategy 1 worked better than Strategy 2 therefore it was selected in our study (see Table 2 in the main text).

	number of genes selected	Naïve RSS	Naïve Correlation	Naïve misclassified subjects (out of 106)	CV RSS	CV Correlation	CV misclassified subjects (out of 106)
**NGSS1, Strategy1**	**41 genes**	**0.884**	**0.935**	**9**	**2.681**	**0.813**	**11**
NGSS1, Strategy2	20 genes	1.725	0.869	17	2.617	0.800	23
**NGSS2, Strategy1**	**13 genes**	**2.549**	**0.800**	**16**	**3.215**	**0.741**	**17**
NGSS2, Strategy2	20 genes	2.606	0.795	16	3.816	0.688	20

In either case, the final predictor is a linear combination of gene expressions, which can be expressed as follows

$$NGSS_j = \hat{\beta}_0 + \sum_i^p X_{ij}\hat{\beta}_i,$$

where $\hat{\beta}_0$ stands for the intercept, X_{ij} is the expression of the ith gene sampled from the jth subject, and $\hat{\beta}_i$ is the linear coefficient corresponding to the ith gene.

These estimates are summarized in Supplementary Tables E1 and E2.

Cross-validation. For both strategies, we evaluate the performance of the fitted models by leave-one-out cross-validations. The residual sum of squares (RSS), Pearson correlation between the actual and predicted GRSS, and prediction accuracy based on the 3.5 cutoff for mild versus severe symptoms, are recorded in these CV studies.

Supplementary Table E2. List of genes used in NGSS1 (Model 2) and the corresponding linear coefficients. Source: "Sig." one of the original 66 significant genes; "Supp.": a supplemental gene.

Gene symbol	Estimated linear coefficient ($\hat{\beta}$)	Marginal p-value	Source
Intercept ($\hat{\beta}_0$)	0.703269912	6.85E-01	-
ST3GAL1	0.059521015	1.21E-03	Sig.
VIM	0.003782824	1.41E-01	Sig.
VCAN	-0.010671636	3.60E-02	Sig.
CXCL2	0.012153063	2.36E-02	Sig.
PTPRC	-0.009655874	1.31E-02	Supp.
FKBP1A	-0.043599137	1.05E-05	Sig.
pk	0.122119918	8.70E-06	Sig.
CCDC80	-0.007741724	2.38E-02	Sig.
HMOX1	-0.02629094	8.67E-02	Sig.
NKG7	-0.001696102	5.20E-01	Sig.
LPXN	0.018386158	2.95E-01	Sig.
PHACTR2	0.069346751	7.53E-03	Sig.
TIA1	-0.033380848	2.83E-02	Sig.
ATP10B	0.027307907	1.97E-01	Sig.
TNFSF10	-0.0018723	2.96E-02	Sig.
INHBA	-0.022899548	3.61E-02	Sig.
MMP19	0.021311619	1.42E-01	Sig.
SMUG1	-0.012079396	2.43E-01	Supp.
MPP1	-0.010764038	2.10E-01	Sig.
RPS15A	0.004696851	8.16E-05	Supp.
CTSL	-0.007780092	7.84E-05	Sig.
HAVCR2	0.073470635	6.43E-06	Sig.
GNS	0.105879463	1.13E-04	Sig.
IL6	0.012406829	2.53E-02	Sig.
SLC39A8	-0.034782958	8.17E-02	Sig.
PTPN7	-0.036920209	1.85E-02	Sig.
RABGAP1L	-0.007262571	2.92E-01	Sig.
SLC7A7	0.02358896	1.27E-01	Sig.
ITGA5	-0.033753825	1.75E-02	Sig.
PPM1M	0.053787695	9.91E-03	Sig.
ARFIP1	0.03076839	1.21E-02	Sig.
MFSD4	-0.007710719	1.34E-01	Sig.
CD163	0.007982385	6.33E-03	Sig.
SHMT2	0.017423769	1.61E-01	Supp.
SERHL2	0.04069629	3.42E-05	Supp.
MAFB	0.080380399	1.53E-02	Sig.

C10orf128	0.037369718	5.12E-02	Sig.
FTLP3	0.012010208	5.11E-02	Sig.
RP11-206L10.8	-0.048018562	8.85E-04	Sig.
N4BP2L2	-0.011088274	1.28E-02	Sig.
VCAN-AS1	-0.014399275	1.31E-01	Sig.

Supplementary Table E3. List of genes used in NGSS2 (Model 4) and the corresponding linear coefficients. Source: "Sig.": one of the 44 original significant genes; "Supp.": a supplemental gene.

Gene symbol	Estimated linear coefficient ($\hat{\beta}$)	Marginal p-value	Source
Intercept ($\hat{\beta}_0$)	5.47173684	9.43E-05	-
EXOSC10	-0.05176355	7.20E-03	Sig.
PPIC	-0.05462252	1.94E-05	Sig.
CCNI	0.01055988	7.07E-06	Sig.
BCKDK	0.06716161	5.04E-04	Sig.
MAP3K13	-0.01209091	1.26E-02	Sig.
MT1G	0.00242952	5.62E-02	Sig.
APOC1	-0.00404016	4.64E-02	Sig.
QTRTD1	-0.04002261	7.18E-03	Sig.
DDRGK1	-0.02822089	2.94E-02	Sig.
SEPHS2	0.07799082	2.65E-03	Sig.
PLK2	0.03691206	2.02E-02	Sig.
CLDN10	0.01013402	4.33E-04	Supp.
PXN	-0.07814369	1.29E-04	Supp.

An Alternative Method Based on CrossNorm

It is known that the choice of normalization procedures can have substantial impact on downstream machine learning algorithms. In addition to using the standard FPKM normalization procedure, we also tried associating GRSS with expression profiles processed by CrossNorm, a normalization procedure designed for processing gene expression data with skewed patterns, and is known to improve the prediction accuracy of downstream machine learning models.(7-10) Below we describe technical details we used for this comparison.

1. The same ComBat procedures used for the FPKM normalized data were applied to CrossNorm processed data.
2. To ensure that transcriptome profiles processed by both FPKM and CrossNorm are directly comparable, the same set of 6,844 genes (filtered by FPKM) were used for both data.
3. For CrossNorm processed data, the initial Pearson correlation test identified 68 genes with significant association with GRSS at FDR<0.05 level. The majority of them (39) were also detected by the FPKM normalized data, which showed that the two normalization methods are largely comparable.
4. Using these 68 genes plus ten supplementary genes identified by PCA as candidate genomic features, we developed Model 2b with the two model selection strategies we used for the FPKM data described earlier. Strategy 1 (bi-directional stepwise model selection based on AIC) selected 31 genes and visit age as informative features. Among them, ten genes were also used in Model 2 build with the FPKM data.
5. Using LOOCV (leave one out cross-validation), we compared Model 2b with Model 2 in terms of CVRSS, correlation, and misclassified subjects. The results are provided in Supplementary Table E4.

Supplementary Table E4. The performance of Model 2b with two model selection strategies. Just like we have seen for Model 2 (NGSS1) trained with FPKM normalized data, Strategy 1 worked better than Strategy 2. Based on this table, we find that Model 2b's performance is slightly worse than that of Model 2 (see Supplementary Table E1) in most comparisons, with the only exception of cross-validated RSS. For convenience, the corresponding results for Model 1 (NGSS1) are provided in this table as well.

	number of genes selected	Naïve RSS	Naïve Correlation	Naïve misclassified subjects (out of 106)	CV RSS	CV Correlation	CV misclassified subjects (out of 106)
Model 2b, Strategy 1	31 genes and visit age	1.259	0.907	16	2.679	0.804	23
Model 2b, Strategy 2	15 genes	2.379	0.815	22	3.331	0.734	28
NGSS1, Strategy1	41 genes	0.884	0.935	9	2.681	0.813	11
NGSS1, Strategy2	20 genes	1.725	0.869	17	2.617	0.800	23

From this table, we find that although Model 2b (with Strategy 1) is slightly more accurate than Model 2 in terms of CVRSS (2.679 versus 2.681), it misclassified significantly more subjects than Model 2 in LOOCV.

References

1. Zou H, Zhang HH. On the Adaptive Elastic-Net with a Diverging Number of Parameters. Ann Stat. 2009;37(4):1733-51. doi: 10.1214/08-AOS625. PubMed PMID: 20445770; PMCID: PMC2864037.
2. Friedman J, Hastie T, Tibshirani R. Regularization Paths for Generalized Linear Models via Coordinate Descent. J Stat Softw. 2010;33(1):1-22. PubMed PMID: 20808728; PMCID: PMC2929880.
3. Belloni A, Chernozhukov V. Least squares after model selection in high-dimensional sparse models. Bernoulli. 2013;19(2):521-47.
4. Liu H, Yu B. Asymptotic properties of Lasso+ mLS and Lasso+ Ridge in sparse high-dimensional linear regression. Electronic Journal of Statistics. 2013;7:3124-69.
5. Lu T, Liang H, Li HZ, Wu HL. High-Dimensional ODEs Coupled With Mixed-Effects Modeling Techniques for Dynamic Gene Regulatory Network Identification. J Am Stat Assoc. 2011;106(496):1242-58. doi: DOI 10.1198/jasa.2011.ap10194. PubMed PMID: ISI:000299662900003.
6. Zou H, Hastie T. Regularization and variable selection via the elastic net (vol B 67, pg 301, 2005). J R Stat Soc B. 2005;67:768-. doi: DOI 10.1111/j.1467-9868.2005.00527.x. PubMed PMID: WOS:000233203400009.
7. Cheng L, Lo LY, Tang NL, Wang D, Leung KS. CrossNorm: a novel normalization strategy for microarray data in cancers. Sci Rep. 2016;6:18898. Epub 2016/01/07. doi: 10.1038/srep18898. PubMed PMID: 26732145; PMCID: PMC4702063.
8. Cheng L, Wang X, Wong PK, Lee KY, Li L, Xu B, Wang D, Leung KS. ICN: a normalization method for gene expression data considering the over-expression of informative genes. Mol Biosyst. 2016;12(10):3057-66. Epub 2016/07/28. doi: 10.1039/c6mb00386a. PubMed PMID: 27452923.
9. Liu X, Li N, Liu S, Wang J, Zhang N, Zheng X, Leung KS, Cheng L. Normalization Methods for the Analysis of Unbalanced Transcriptome Data: A Review. Front Bioeng Biotechnol. 2019;7:358. Epub 2020/02/11. doi: 10.3389/fbioe.2019.00358. PubMed PMID: 32039167; PMCID: PMC6988798.
10. Liu X, Zheng X, Wang J, Zhang N, Leung KS, Ye X, Cheng L. A long non-coding RNA signature for diagnostic prediction of sepsis upon ICU admission. Clinical and translational medicine. 2020;10(3).
